# An evaluation of the inhibitory effects against rotavirus infection of edible plant extracts

**DOI:** 10.1186/1743-422X-9-137

**Published:** 2012-07-26

**Authors:** Karen Knipping, Johan Garssen, Belinda van’t Land

**Affiliations:** 1 Danone Research, Centre for Specialised Nutrition, P.O. Box 7005, 6700, CA, Wageningen, The Netherlands; 2 Utrecht Institute for Pharmaceutical Sciences, P.O. Box 80082, 3508, TB, Utrecht, The Netherlands; 3 Department of Pediatrics, Wilhelmina Children’s Hospital, University Medical Center, P.O. Box 85090, 3508, AB, Utrecht, The Netherlands

**Keywords:** Rotavirus, Antiviral activity, Transepithelial resistance, Plant extracts

## Abstract

**Background:**

Rotaviruses are the single most important cause of severe diarrhea in young children worldwide. The developments of specific, potent and accessible antiviral treatments that restrain rotavirus infection remain important to control rotavirus disease.

**Methods:**

150 plant extracts with nutritional applications were screened *in vitro* on MA-104 cells for their antiviral activity against rhesus rotavirus (RRV). One extract (*Aspalathus linearis* (Burm.f.) R.Dahlgren) was also tested for its effect on the loss of transepithelial resistance (TER) of Caco-2 cells caused by simian rotavirus (SA-11) infection.

**Results:**

Aqueous extracts of *Nelumbo nucifera* Gaertn. fruit, *Urtica dioica* L. root, *Aspalathus linearis* (Burm.f.) R.Dahlgren leaves, *Glycyrrhiza glabra* L. root and *Olea europaea* L. leaves were found to have strong significant antiviral activity with a 50% inhibitory concentration (IC50) < 300 μg/ml. The pure compound 18ß-glycyrrhetinic acid from *Glycyrrhiza glabra* was found to have the strongest antiviral activity (IC50 46 μM), followed by luteolin and vitexin from *Aspalathus linearis* (IC50 respectively 116 μM and 129 μM) and apigenin-7-O-glucoside from *Melissa officinalis* (IC50 150 μM). A combination of *Glycyrrhiza glabra* L. + *Nelumbo nucifera* Gaertn. and *Urtica dioica L.* + *Nelumbo nucifera* Gaertn. showed synergy in their anti-viral activities. *Aspalathus linearis* (Burm.f.) R.Dahlgren showed no positive effect on the maintenance of the TER.

**Conclusions:**

These results indicate that nutritional intervention with extracts of *Nelumbo nucifera* Gaertn., *Aspalathus linearis* (Burm.f.) R.Dahlgren, *Urtica dioica* L., *Glycyrrhiza glabra* L. and *Olea europaea* L. might be useful in the treatment of diarrhea caused by rotavirus infection.

## Background

Rotavirus is still one of the leading causes of severe dehydrating diarrhea in children under the age of five and causes the deaths of 453,000 children younger than 5 years annually [1]. Rotaviruses, belonging to a genus of double-stranded RNA viruses in the family Reoviridae, infect the mature villous epithelial cells of the small intestine, often leading to fever, vomiting, and diarrhea in children. For the treatment of rotavirus gastroenteritis, intravenous fluid administration has been used successfully in treating only the direct consequences of the dehydration from diarrhea. Gastrogard-R® is a prophylactic treatment of ‘at risk’ children aged one month to three years to prevent diarrhea due to rotavirus infection and the efficacy of treatment was established in a clinical trial in children aged 3 to 15 months [2]. Gastrogard-R® is prepared from the colostrum of hyperimmunised cows and contains immunoglobulins against serotypes G1 and G3 of the human rotavirus. We have demonstrated in an *in vivo* rotavirus infection mouse model that Gastrogard-R® is able to completely inhibit rotavirus-induced diarrhea in suckling mice [3]. A first generation of licensed rotavirus vaccine was withdrawn from the market a year after introduction due to a possible correlation between vaccine application and the occurrence of intussusceptions [4]. Two live-attenuated vaccines have been licensed recently and have so far proven safe and highly efficacious in developed countries. In developing countries, clinical trials are being undertaken and early results found that the vaccine significantly reduces severe diarrhea episodes due to rotavirus (48.3% for Asia and 30.2% for Africa) [5]. However, rotavirus vaccines are expensive and may not be affordable for the developing world at present, compromising full vaccine coverage. This has reinforced the need to develop alternative approaches to control rotavirus disease. Natural compounds have been identified as ideal candidates for antiviral drugs because they are cheaper and effective [6]. To date, many natural compounds have known antirotavirus effects in clinical studies [7-9], in animal experiments [10] and *in vitro*[11-14]. In the current study we investigated 150 edible plant extracts and some of their natural compounds for *in vitro* anti-rotavirus infection effects.

MA104 cells is an established cell line for rotavirus infections studies on which rotavirus receptors have been identified on the cells [15]. Trypsin usually is added during infection of MA104 cells with rotavirus to increase the infectivity [16-18]. Intestinal epithelial permeability studies are often performed with human Caco-2 cells [19]. These cells develop polarization of distinct apical and basolateral surfaces separated by tight junctions at areas of cell-to-cell contact closely resembling the physiological situation in the intestine. The transepithelial resistance (TER) is a measure for the tightness of the tight junctions formed by Caco-2 cells. Rotaviruses are known to infect epithelial cells of the small intestine and *in vitro* rotavirus infection of Caco-2 cells caused disruption of tight junctions and loss of TER in the absence of cell death [20]. To investigate whether the loss of TER could be prevented, Caco-2 cells were infected with SA-11 rotavirus in the presence of hyperimmune colostrum (Gastrogard-R®), plant extracts or pure compounds. From both models, the results of 150 aqueous plant extracts on their antiviral action against rotavirus are presented in a search for novel agents for the treatment of human diarrhea caused by rotavirus infection.

## Material and methods

### Cells and viruses

MA104 cells (African green monkey kidney cells; ECACC, Salisbury, UK 85102918) and Caco-2 cells (Human Caucasian colon adenocarcinoma; ECACC) were maintained in Eagle’s minimal essential medium (MEM; Life Technologies, Breda, The Netherlands) supplemented with 10% fetal calf serum (FCS; Greiner Bio-one, Alphen a/d Rijn, The Netherlands), 2 mM Sodium pyruvate (Life Technologies) and 1% non-essential amino acids (Life Technologies). The viruses selected for this study were the simian rotavirus SA-11 strain (ATCC) and the rhesus rotavirus (RRV) strain, kindly provided by Dr. Richard Ward, Cincinnati Children's Hospital Medical Center, USA. The viruses were grown in MA104 cells and concentrated by ultracentrifugation. The titers were determined using a titration assay in MA104 cells resulting in a 50% cell culture infective dose (CCID50) of 1x10^8^ of both SA-11 and RRV.

### Hyperimmune colostrum (Gastrogard-R®), plant extracts and pure compounds

Hyperimmune colostrum (HIC) (Gastrogard-R®, Nutricia, Adelaide, Australia) was tested in the titration assay on MA104 cells in the concentrations of 1-2-5-10-50-100 μg/ml medium and in the transepithelial resistance measurement in the concentrations of 50 and 100 μg/ml medium. 150 Plant extracts with known nutritional applications were selected from an ‘in house’ herbal extract collection (purchased from PhytoMyco Research Pvt. Ltd., Mysore District, Karnataka State, India) consisting of 10,000 herbal extracts. In a set-up for high throughput screening, all plant extracts were tested as a first screening in an antiviral titration assay with MA104 cells in the concentrations of 400 and 500 μg/ml in medium. When an inhibition of infection was determined (<20% inhibition), a 50% inhibitory concentration (IC50) value was calculated. Extracts that reduced the rotavirus infection were studied further in two concentrations close to the calculated IC50. Extracts unable to inhibit the infection were appointed an arbitrary IC50 of 1000 μg/ml. A complete list of tested herbal extracts is listed in Additional file 1. Synergy between two plant extracts was investigated in the titration assay by assaying the separate compounds as well as combinations hereof on the same plate. The separate compounds were tested in the calculated IC50 concentration whereas the combination a mixture was of the IC25 concentration of both extracts. 18ß-glycyrrhetinic acid (Sigma-Aldrich, St. Louis, USA) was tested in the concentrations of 10-20-30-40-50-100 μM in medium. Luteolin, vitexin and apigenin-7-O-glucoside (Indofine Chemical Company, Hillsborough, USA) were tested in the concentrations of 50 and 100 μM in medium. A complete list of tested compounds is listed in Additional file 2.

### Antiviral titration assay

Confluent MA104 cells in a 96-wells plate were first incubated with various concentrations of the plant extract and then a two-fold titration range of RRV, starting at 1*10^6^ CCID50, was added in the presence of 0.5 mg trypsin/ml (Life Technologies). One well with MA104 cells was incubated with the highest concentration of the extract as a control. Infection of MA104 cells with rotavirus induces syncytium after 3-4 days, which is visible through microscopy. A titration of RRV on MA104 was used as a measure of maximum infection and the sum of infected wells was considered 100% infection. Inhibition of the plant extract was calculated against the maximum infection. Maximum of infection and each concentration of the plant extract were tested in duplicate.

### Transepithelial resistance (TER) measurement

The TER is a measure for the tightness of the tight junctions formed by Caco-2 cells. One electrode of the epithelial volt-ohm meter (World Precision Instuments, Sarasota, USA) was placed into the apical compartment of a transwell system (Corning Life Sciences, New Jersey, USA) and the other into the basolateral compartment. The electrical resistance is then measured across the cell monolayer after the passage of a defined current pulse. Experiments were carried out at two weeks post confluency with fully differentiated cells. One day before infection, the Caco-2 cells were cultured in medium without FCS. Prior to infection, the SA-11 rotavirus inoculum was activated for 20 min by treatment with 0.5 mg trypsin/ml (Invitrogen). The Caco-2 cells were apically infected with an inoculum of activated rotavirus (1*10^6^ CCID50) in the presence of the extract and incubated at 37°C and 5% CO_2_. The integrity of the confluent polarized monolayer was checked by measuring TER with a volt-ohmmeter. TER (in units of ohm times centimeters squared) was calculated as the measured electrical resistance times the surface area of a filter. The background reading of a control filter was measured in every experiment. Infections were carried out in triplicates and all measurements were done at various time points post infection. The permeability of Caco-2 cell monolayer was also determined by measuring the paracellular passage of HRP or FITC-dextran from apical to the basolateral compartments of the culture chamber. The concentrations of FITC-dextran were determined by measuring the fluorescence at λ_excitation_ 485 nm and λ_emission_ 520 nm. The concentration of HRP was determined by incubating 50 μl of the sample with 50 μl TMB for 10 min at RT. The reaction was stopped with 50 μl H_2_SO_4_ and the absorbance measured at 450 nm.

### Analysis

The 50% cell culture infective dose (CCID50) of SA-11 and RRV were calculated according the Reed and Muench statistical method [21]. 50% inhibitory concentration (IC50) values were determined by calculating a statistically validated non-linear regression curve through the plotted acquired values. X-values (i.e. dosage) corresponding to the maximal y-value divided by 2, are calculated from the mathematical regression curve formula.

## Results

### Hyperimmune colostrum (Gastrogard-R®)

**Figure 1:**
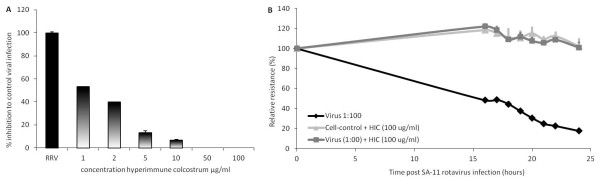
**A Inhibitory potential of hyperimmune colostrum (HIC) (Gastrogard-R®) on RRV infection of MA104 cells.** Maximum infection with RRV was set to 100% and the inhibition of HIC in the concentrations 1-2-5-10-50-100 μg/ml was calculated as a percentage against maximum infection. Bars represent average of triplicate values + SD. **B** Maintenance of transepithelial resistance (TER) ± SD of Caco-2 cells by HIC after infection with SA-11 rotavirus. The black line shows the decline in relative resistance in time caused by the SA-11 rotavirus infection. The light grey line depicts the cell control with no infection but in the presence of HIC (100 μg/ml). The dark grey line depicts infection of Caco-2 cells with SA-11 in the presence of HIC (100 μg/ml).

The hyperimmune colostrum (HIC) is used as a positive control and therefore tested in the antiviral titration assay with MA104 cells in several concentrations (Figure 1A). The concentration of 1 μg/ml showed an inhibition of infection with 50% compared to the maximum infection with RRV.

The effect of two rotavirus strains on the transepithelial resistance (TER) with Caco-2 cells was first determined. Only the SA-11 rotavirus strain, and not RRV, showed a decrease in TER. An infection of SA-11 resulted also in a progressive increase in the paracellular permeability to HRP (a 40 kD molecule) and FITC-dextran (a 4 kD molecule) in correlation to the time post infection (data not shown). The decline in TER of Caco-2 cells by SA-11 and the effect of the HIC (100 μg/ml) on the TER during SA-11 infection is depicted in Figure 1B. Infection of Caco-2 cells with SA-11 results in a decline of the TER for more than 80%. Infection in the presence of HIC showed a complete maintenance of the TER, comparable to non-infected Caco-2 cells in the presence of HIC.

### Screening of plant extracts

**Figure 2:**
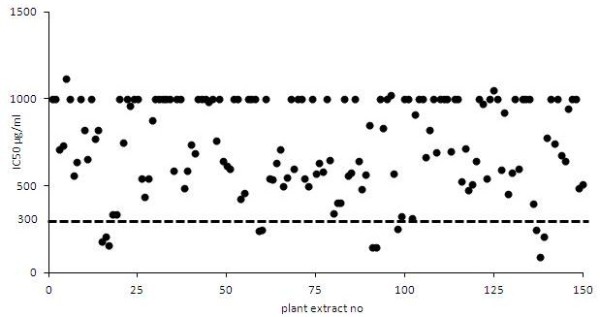
Of 150 plant extracts (black dots), 11 extracts strongly inhibited the rotavirus infection of MA104 cells with a 50% inhibitory concentration (IC50) <300 μg/ml (below the black dashed line).

**Figure 3:**
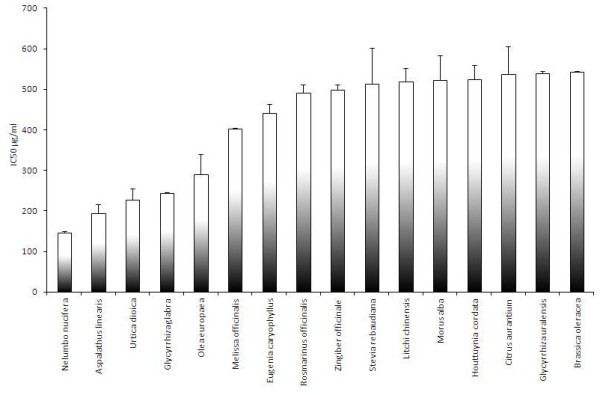
**Five strong (IC50 <300 μg/ml) and eleven moderate (IC50 between 300 and 550 μg/ml) inhibitory plant extract.** Bars represent average of duplicate analysis + SD.

A study of 150 edible plant extracts dissolved in medium was performed to investigate their inhibitory effects on rotavirus infection of MA104 cells (Figure 2). Eleven extracts were able to inhibit the rotavirus infection with a 50% inhibitory concentration (IC50) <300 μg/ml. Among these 11 extracts were 3 different Urtica dioica L., 2 different Nelumbo nucifera Gaertn., 3 different Aspalathus linearis (Burm.f.) R.Dahlgren, 2 different Glycyrrhiza glabra L. extracts and one Olea europeae L. extract. The most potent extract of each plant together with eleven moderate (IC50 between 300 and 550 μg/ml) inhibitors of rotavirus infection are depicted in Figure 3. Aspalathus linearis (Burm.f.) R.Dahlgren has also been tested for its effect on the TER (100 and 200 μg/ml), but no positive effect on the maintenance of the TER was measured.

### Screening of pure compounds

Twenty-four pure compounds, known to be present in the strong and moderate inhibitory plant extracts, have been tested in the antiviral titration assay in the concentrations of 50 and 100 μM. Only four compounds showed an inhibitory effect, 18ß-glycyrrhetinic acid from *Glycyrrhiza glabra* was found to have the strongest antiviral activity (IC50 46 μM), followed by luteolin and vitexin from *Aspalathus linearis* (IC50 respectively 116 μM and 129 μM) and apigenin-7-O-glucoside from *Melissa officinalis* (IC50 150 μM). 18ß-glycyrrhetinic has also been tested for its effect on the TER (25-30-40-50 μg/ml), but no positive effect on the maintenance of the TER was measured.

### Synergy between plant extracts

**Figure 4:**
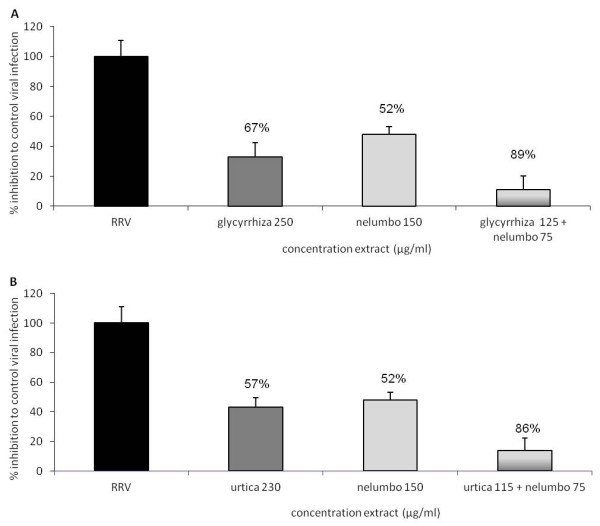
**Synergism in antiviral activity in the combinations*Glycyrrhiza glabra*L. +*Nelumbo nucifera*Gaertn. A and*Urtica dioica*L. +*Nelumbo nucifera*Gaertn. B**. The separate extracts were tested in their IC50 concentration, combinations were made of the IC25 concentration of the extracts. Inhibition was calculated against the control viral infection (RRV; 100%). Bars represent average of triplicate values + SD.

Synergism is the phenomenon in which the combined action of two or more extracts is greater than the sum of the individual effects of each extract. Two combinations of plant extracts showed synergy and are depicted in Figure 4. *Glycyrrhiza glabra* L. (250 μg/ml) inhibited infection with 67% and *Nelumbo nucifera* Gaertn. (150 μg/ml) with 52%, whereas a combination of the IC25 (Glycyrrhiza 125 μg/ml and Nelumbo 75 μg/ml) showed an inhibition of 89%. This is also seen with a combination of *Urtica dioica* L. and *Nelumbo nucifera* Gaertn. *Urtica dioica* L. (230 μg/ml) inhibited infection with 57% and *Nelumbo nucifera* Gaertn. (150 μg/ml) with 52%. The combination of Urtica 115 μg/ml and Nelumbo 75 μg/ml showed an inhibition of 86% of the infection.

## Discussion

Recently, there is a growing interest in the interaction between pharmacology and nutrition science. Pharmaceuticals are generally developed to treat, cure or prevent disease and the primary goal of nutrition is to maintain or even improve health. This does not imply that there is no role for nutrition in preventing or curing disease [22]. The 150 plant extracts used in the screening here were selected because of their known nutritional uses. The nutritional applications and antiviral activities of the five strongest rotavirus inhibitors are discussed. *Nelumbo nucifera* (sacred water lotus) is not a well known food ingredient in Europe, but in Asia the roots are often eaten as a vegetable in soups, deep-fried, stir-fried and braised dishes. *Nelumbo nucifera* is known to have antiviral action against HIV [23] and Herpes simplex virus (HSV) [24]. *Aspalathus linearis* leaves are used to make redbush tea which has been popular in Southern Africa for generations and is now consumed in many countries. No antiviral activity has been reported yet for *Aspalathus linearis*. *Urtica dioica* (stinging nettle) can be used in a variety of recipes and nettle soup is a common use of the plant, particularly in Northern and Eastern Europe. In Nepal and Northern India it is a very popular vegetable and cooked with Indian spices. Known antiviral actions of *Urtica dioica* are inhibition of both HIV and FIV, the feline variant of HIV [25,26]. *Glycyrrhiza glabra* is also known as European licorice and the root extract is used as flavoring in sweets, baked goods, ice cream and soft drinks. Only one other member of the *Glycyrrhiza* sp., *Glycyrrhiza uralensis* (Chinese licorice) was already know from literature to have antiviral activity against rotavirus [27]. *Glycyrrhiza glabra* itself is known to have inhibitory activity against several unrelated DNA and RNA viruses [28-30]. *Olea europeae* fruit (olive) is of major agricultural importance in the Mediterranean region as the source of olive oil. *Olea europeae* exhibits antiviral activity against viral haemorrhagic septicaemia rhabdovirus (VHSV) [31]. For these nutritional ingredients we have demonstrated antirotavirus activity *in vitro*. This illustrates the potential for the use of specific plant extracts for treatment or prevention of rotavirus illness.

Twenty-four pure compounds, known to be present in the strong and moderate rotavirus inhibitory plant extracts, have been tested in the antiviral titration assay, but only 4 compounds showed antiviral activity. 18ß-glycyrrhetinic acid, a constituent of *Glycyrrhiza glabra*, was the strongest inhibitor. Glycyrrhizic acid is one of the bioactive compounds of licorice roots and is composed of one molecule of 18ß-glycyrrhetinic acid, which has a steroid-like structure, and two molecules of glucuronic acid. Lin et al. reported 18ß-glycyrrhetinic acid to have a stronger action against Epstein-Barr virus than its parental compound glycyrrhizic acid [32]. Two constituents of *Aspalathus linearis*, luteolin and vitexin, also showed antiviral activity against rotavirus in the antiviral titration assay. Luteolin is a flavonoid most often found in leaves and vitexin is an apigenin flavone glucoside. Both compounds are found in many different plant species other than *Aspalathus*. The weakest inhibitor was apigenin 7-O-glucoside, also a flavonoid and can be found in for example lemon balm (*Melissa officinalis*), chamomile (*Chamomilla recutita*) and celery (*Apium graveolens*). So far as we know this is the first study showing that luteolin, vitexin and apigenin 7-O-glucoside have antiviral activity *in vitro*. Although the mechanism of action has yet to be defined, this is a first step in a potential wider, more pharmacological use of these nutritional ingredients. The possibility that there are other active compounds present in the positive plant extracts has to be further investigated. The positive control hyperimmune colostrum (HIC) showed inhibitory action against rotavirus infection in both the antiviral titration assay with RRV in MA104 cells as well as in maintaining the TER in Caco-2 cells infected with SA-11. In synergism studies, where the extracts have been combined in the IC25 concentration, synergism in antiviral activity was found in the combinations *Glycyrrhiza glabra* L. + *Nelumbo nucifera* Gaertn. and *Urtica dioica* L. + *Nelumbo nucifera* Gaertn. The separate extracts with IC50 concentration showed an inhibition of infection of about 50%. Both combinations in the IC25 concentration of the extracts showed an inhibition of more than 50%, respectively 89% and 86%. This indicates that the extracts in the combination have different antiviral actions. This illustrates the importance for further research into the mechanism of action of the individual ingredients, as well as *in vivo* validation of the antiviral capacity.

The level of antiviral activity as tested *in vitro* can only be used as an indication for the therapeutical benefit of the component *in vivo*. Within this study, the aspects related to toxicity, taste, stability, availability, duration of supplementation, regulation, drug interactions and pharmacokinetic profile were not taken into account. Therefore, no statements can currently be made regarding the *in vivo* applicability of any of these nutritional compounds. However, since most herbs are currently used as nutritional ingredients and rotavirus is an intestinal pathogen, there is clear potential for the interventions as described.

Within several studies, natural extracts were tested for their antiviral capacity [11,33,34]. Although the isolated active compounds have shown antiviral activities with IC50 values ranging from 0.1 mg/ml – 250 mg/ml, the crude extract most of the times only reached an antiviral activity IC50 was not below 30 mg/ml. Therefore a crude extract reaching the IC50 of <0.3 mg/ml was regarded as a strong inhibitor. Moreover, it is specified that each extract and active compound has its own bioavailability and activity *in vivo*[35]. Therefore additional research in this field is needed on the potential of use for these natural compounds single or in combinations of each other for the use in clinical setting. Comparisons and screening are therefore a valuable start.

The titration assay using MA104-cells has proven to be a useful assay to measure the inhibitory ability of plant extracts on a rotaviral infection. In contrast, assessment of TER did not reveal inhibitory compounds. In this study only the SA-11 rotavirus strain, and not RRV, showed a decrease in TER even though previous published data has shown an effect of RRV on Caco-2 TER [20]. Cho et all. showed that absorption enhancers like 18ß-glycyrrhetinic acid and taurine can decrease TER of Caco-2 cells and increase the permeability in a dose-dependent and time-dependent manner [36]. These results indicate that absorption enhancers can widen the tight junction even without rotavirus infection. In future experiments using TER with extracts, this increase in permeability without rotavirus infection should be excluded for each extract.

## Conclusions

These data demonstrate for the first time that generally used nutritional extracts of *Nelumbo nucifera* Gaertn., *Aspalathus linearis* (Burm.f.) R.Dahlgren, *Urtica dioica L.*, *Glycyrrhiza glabra* L. and *Olea europaea* L. have *in vitro* antiviral activity against rotavirus. None of these plants have been reported before as having antiviral action against rotaviruses. The antiviral action can even be enhanced by combining of *Glycyrrhiza glabra* L., *Nelumbo nucifera* Gaertn. and/or *Urtica dioica* L. extracts, indicating different mechanisms of action. Therefore, combinations of these plants are potentially useful in the treatment of diarrhea caused by rotavirus.

## Abbreviations

SA-11, simian rotavirus; RRV, rhesus rotavirus; IC50, 50% inhibitory concentration; CCID50, 50% cell culture infective dose.

## Competing interests

The authors are employees of Danone Research - Centre for Specialised Nutrition, but declare that they have no competing interests.

## Authors' contributions

KK participated in the design, execution and interpretation of the *in vitro* experiments and drafted the manuscript. JG has been involved in drafting the manuscript for important intellectual content. BvtL participated in the design and interpretation of the experiments and helped to draft the manuscript. All authors read and approved the final manuscript.

## Supplementary Material

Additional file 1 **Appendix 1.** List of tested herbal extracts. Click here for file

Additional file 2 **Appendix 2.** List of tested pure components. Click here for file
