# Supplementary material for: An evaluation of the inhibitory effects against rotavirus infection of edible plant extracts
Source: Virol J. 2012 Jul 26;9:137. doi: 10.1186/1743-422X-9-137 (PMC3439294; doi:10.1186/1743-422X-9-137)
Supplement: Additional file 1 — Appendix 1. List of tested herbal extracts. [file 1743-422X-9-137-S1.doc]

Appendix 1. List of tested herbal extracts

| **Plant** | **Family** | **Common name** | **Plant part** | **UIN** | **Virus** | **Tested conc. µg/ml** | **IC50 µg/ml** |
| --- | --- | --- | --- | --- | --- | --- | --- |
| *Agastache rugosa* Kuntze | Lamiaceae | korean mint | herb | 11768 | RRV | 200-300 | - |
| *Allium ascalonicum* L. | Alliaceae | shallot | bulb | 12145 | RRV | 200-300 | - |
| *Allium sativum* L. var. *vulgare* | Alliaceae | garlic | unknown | 1856 | RRV | 200-300 | 706,9 |
| *Allium sativum* L. var. *vulgare* | Alliaceae | garlic | bulb | 2165 | RRV | 200-300 | 733,1 |
| *Alpinia galanga* ( L. ) Willd. | Zingiberaceae | greater galanga | root | 12058 | RRV | 400-500 | 1114,9 |
| *Ananas comosus* ( L. ) Merr. | Bromeliaceae | pineapple | dried fruit | 776 | RRV | 25-50-100 | toxic |
| *Ananas comosus* ( L. ) Merr. | Bromeliaceae | pineapple | freeze dried fruit | 922 | RRV | 25-50-100 | - |
| *Ananas comosus* ( L. ) Merr. | Bromeliaceae | pineapple | juice chrystals | 1805 | RRV | 200-300 | 558,9 |
| *Ananas comosus* ( L. ) Merr. | Bromeliaceae | pineapple | juice chrystals | 1805 | RRV | 400-500 | 639,2 |
| *Annona squamosa* L. | Annonaceae | sugar apple | fruit/leaf | 12062 | RRV | 200-300 | - |
| *Arctium lappa* L. | Asteraceae | burdock, gobo | unknown | 805 | RRV | 400-500 | 821,0 |
| *Arctium lappa* L. | Asteraceae | burdock, gobo | root | 2179 | RRV | 400-500 | 654,2 |
| *Arctium lappa* L. | Asteraceae | burdock, gobo | root | 2179 | SA11 | 300-400-500 | - |
| *Arctium lappa* L. | Asteraceae | burdock, gobo | root | 8525 | RRV | 200-300 | 771,7 |
| *Arctium lappa* L. | Asteraceae | burdock, gobo | fruit | 8658 | RRV | 200-300 | toxic |
| *Arctium lappa* L. | Asteraceae | burdock, gobo | root | 12223 | RRV | 200-300 | 819,3 |
| *Arctium lappa* L. | Asteraceae | burdock, gobo | whole plant | 15737 | RRV | 25-50 | toxic |
| *Aspalathus linearis* ( Burm.f. ) R.Dahlgren | Leguminosae | redbush tea | leaves | 1831 | RRV | 100-150 | 180,0 |
| *Aspalathus linearis* ( Burm.f. ) R.Dahlgren | Leguminosae | redbush tea | leaves | 1831 | RRV | 100-200 | 210,3 |
| *Aspalathus linearis* ( Burm.f. ) R.Dahlgren | Leguminosae | redbush tea | leaves | 1831 | SA11 | 100-150-200 | 156,4 |
| *Aspalathus linearis* ( Burm.f. ) R.Dahlgren | Leguminosae | redbush tea | herb | 1890 | RRV | 100-200 | 338,4 |
| *Aspalathus linearis* ( Burm.f. ) R.Dahlgren | Leguminosae | redbush tea | herb | 1890 | RRV | 100-200 | 335,6 |
| *Aspalathus linearis* ( Burm.f. ) R.Dahlgren | Leguminosae | redbush tea | unknown | 2188 | RRV | 100-200 | - |
| *Aspalathus linearis* ( Burm.f. ) R.Dahlgren | Leguminosae | redbush tea | leaves | 2191 | RRV | 400-500 | 750,0 |
| *Berberis aristata* DC. | Berberidaceae | tree turmeric | unknown | 159 | RRV | 200-300 | - |
| *Berberis aristata* DC. | Berberidaceae | tree turmeric | unknown | 2187 | RRV | 200-300 | 957,9 |
| *Beta vulgaris L.* ssp. *vulgaris* var. *ruba* | Chenopodiaceae | beet | fruit | 25534 | RRV | 400-500 | - |
| *Beta vulgaris L.* ssp. *vulgaris* var. *ruba* | Chenopodiaceae | beet | fruit | 25534 | SA11 | 300-400-500 | - |
| *Brassica oleracea* L. var. *italica* | Brassicaceae | broccoli | flower | 1118 | RRV | 400-500 | 542,7 |
| *Brassica oleracea* L. var. *italica* | Brassicaceae | broccoli | flower | 1118 | RRV | 400-500 | 544,4 |
| *Brassica oleracea* L. var. *italica* | Brassicaceae | broccoli | flower | 1118 | SA11 | 300-400-500 | 436,0 |
| *Brassica oleracea* L. var. *italica* | Brassicaceae | broccoli | leaves | 2166 | RRV | 200-300 | 877,9 |
| *Calendula officinalis* L. | Asteraceae | pot marigold | flower | 8526 | RRV | 200-300 | toxic |
| *Camellia sinensis* ( L. ) Kuntze | Theaceae | green tea | leaves | 1092 | RRV | 200-300 | toxic |
| *Camellia sinensis* ( L. ) Kuntze | Theaceae | green tea | leaves | 1893 | RRV | 200-300 | toxic |
| *Capsicum frutescens* L. | Solanaceae | cayenne | fruit | 12137 | RRV | 200-300 | - |
| *Centella asiatica* ( L. ) Urb. | Apiaceae | gotu kola | herb | 127 | RRV | 200-300 | - |
| *Centella asiatica* ( L. ) Urb. | Apiaceae | gotu kola | leaf/stem | 25705 | RRV | 200-300 | - |
| *Chrysanthemum morifolium* Ramat. | Asteraceae | chrysant | flower | 5 | RRV | 400-500 | - |
| *Chrysanthemum morifolium* Ramat. | Asteraceae | chrysant | flower | 5 | SA11 | 300-400-500 | - |
| *Chrysanthemum morifolium* Ramat. | Asteraceae | chrysant | flower | 37 | RRV | 200-300 | 585,5 |
| *Citrus aurantiifolia* (Christm.) Swingle | Rutaceae | lime | fruit | 1899 | RRV | 400-500 | - |
| *Citrus aurantium* var. *amara* | Rutaceae | bitter orange | fruit | 741 | RRV | 400-500 | - |
| *Citrus aurantium* var. *amara* | Rutaceae | bitter orange | fruit | 843 | RRV | 400-500 | 489,0 |
| *Citrus aurantium* var. *amara* | Rutaceae | bitter orange | fruit | 843 | RRV | 400-500 | 586,2 |
| *Citrus aurantium* var. *amara* | Rutaceae | bitter orange | fruit | 2017 | RRV | 400-500 | 684,2 |
| *Citrus aurantium* var. *amara* | Rutaceae | bitter orange | fruit | 8092 | RRV | 400-500 | 738,8 |
| *Citrus aurantium* var. *amara* | Rutaceae | bitter orange | fruit | 11831 | RRV | 400-500 | - |
| *Citrus limon* ( L. ) Burm.f. | Rutaceae | lemon | fruit powder | 1797 | RRV | 400-500 | - |
| *Citrus medica* L. | Rutaceae | citron | fruit | 8212 | RRV | 400-500 | - |
| *Citrus nobilis* Lour. | Rutaceae | tangerin | plant | 12053 | RRV | 400-500 | 980,8 |
| *Citrus paradisi* Macfad. | Rutaceae | grapefruit | fruit | 1791 | RRV | 400-500 | - |
| *Citrus reticulata* Blanco | Rutaceae | mandarin | peel | 8094 | RRV | 400-500 | 761,0 |
| *Citrus sinensis ( L. )* Osbeck | Rutaceae | sweet orange | fruit | 555 | RRV | 400-500 | - |
| *Crataegus pinnatifida* Bunge | Rosaceae | hawthorne | leaves | 2092 | RRV | 400-500 | 641,0 |
| *Curcuma longa* L. | Zingiberaceae | turmeric/curcumin | rhizome powder | 866 | RRV | 10-25-50-100-200 | 614,4 |
| *Curcuma longa* L. | Zingiberaceae | turmeric/curcumin | rhizome powder | 866 | RRV | 100-200-300 | 595,8 |
| *Cydonia oblonga* Mill. | Rosaceae | quince | seed | 12087 | RRV | 400-500 | contaminated |
| *Eleutherococcus senticosus* (Rupr. & Maxim.) Maxim. | Araliaceae | chinese ginseng | rhizome | 149 | RRV | 400-500 | - |
| *Eugenia caryophyllus* ( Spreng. ) Bullock & S.G.Harrison | Myrtaceae | clove | flower | 8686 | RRV | 400-500 | - |
| *Eugenia caryophyllus* ( Spreng. ) Bullock & S.G.Harrison | Myrtaceae | clove | flower | 12198 | RRV | 400-500 | 426,1 |
| *Eugenia caryophyllus* ( Spreng. ) Bullock & S.G.Harrison | Myrtaceae | clove | flower | 12198 | RRV | 300-400-500 | 456,8 |
| *Gentiana lutea* L. | Gentianaceae | yellow gentian | root | 8521 | RRV | 400-500 | - |
| *Glycine max* ( L. ) Merr. | Leguminosae | soy | seed | 8287 | RRV | 400-500 | - |
| *Glycine max* ( L. ) Merr. | Leguminosae | soy | fruit | 8593 | RRV | 400-500 | - |
| *Glycyrrhiza glabra* L. | Leguminosae | licorice | root | 238 | RRV | 10-25-50-100-200 | 243,5 |
| *Glycyrrhiza glabra* L. | Leguminosae | licorice | root | 238 | RRV | 100-200-300 | 244,8 |
| *Glycyrrhiza glabra* L. | Leguminosae | licorice | root | 238 | SA11 | 300 | - |
| *Glycyrrhiza uralensis* Fisch. ex DC. | Leguminosae | chinese licorice | root | 8308 | RRV | 400-500 | 543,5 |
| *Glycyrrhiza uralensis* Fisch. ex DC. | Leguminosae | chinese licorice | root | 8308 | RRV | 400-500 | 536,6 |
| *Helichrysum italicum* ( Roth ) G.Don | Asteraceae | curry plant | flower | 25190 | RRV | 400-500 | 632,4 |
| *Hericium erinaceus* Bull. | Hericiaceae | bearded tooth | unknown | 2247 | RRV | 400-500 | 709,2 |
| *Houttuynia cordata* Thunb. | Saururaceae | chameleon plant | herb | 8454 | RRV | 400-500 | 500,0 |
| *Houttuynia cordata* Thunb. | Saururaceae | chameleon plant | herb | 8454 | RRV | 400-500 | 548,8 |
| *Humulus lupulus* L. | Cannabaceae | hop | resin | 2282 | RRV | 400-500 | toxic |
| *Humulus lupulus* L. | Cannabaceae | hop | resin | 2282 | RRV | 200-300 | toxic |
| *Hyssopus officinalis* L. | Lamiaceae | hyssop | flower | 1068 | RRV | 400-500 | - |
| *Hyssopus officinalis* L. | Lamiaceae | hyssop | herb | 12230 | RRV | 400-500 | 600,1 |
| *Juniperus communis* L. | Cupressaceae | common juniper | fruit | 12237 | RRV | 400-500 | toxic |
| *Juniperus communis* L. | Cupressaceae | common juniper | fruit essential oil | 25466 | RRV | 400-500 | - |
| *Larrea tridentata* Coville | Zygophyllaceae | creosote bush | herb | 25905 | RRV | 400-500 | - |
| *Litchi chinensis* Sonn. | Sapindaceae | lychee | seed | 8146 | RRV | 400-500 | 543,1 |
| *Litchi chinensis* Sonn. | Sapindaceae | lychee | seed | 8146 | RRV | 400-500 | 496,0 |
| *Litchi chinensis* Sonn. | Sapindaceae | lychee | seed | 11775 | RRV | 400-500 | toxic |
| *Lomatium dissectum* ( Nutt. ex Torr. & A.Gray ) Mathias & Constance | Apiaceae | fernleaf biscuitroot | root | 25911 | RRV | 400-500 | toxic |
| *Lonicera japonica* Thunb. | Caprifoliaceae | japanese honeysuckle | flower | 487 | RRV | 400-500 | - |
| *Luffa cylindrica* ( L.) M.Roem. | Cucurbitaceae | smooth sponge gourd | retinervus | 8198 | RRV | 400-500 | 567,7 |
| *Lycopersicon esculentum* Mill. | Solanaceae | tomato | fruit | 836 | RRV | 400-500 | 629,2 |
| *Lycopersicon esculentum* Mill. | Solanaceae | tomato | fruit | 2285 | RRV | 400-500 | 579,2 |
| *Malus domestica* Borkh. | Rosaceae | apple | fruit | 740 | RRV | 400-500 | toxic |
| *Malus domestica* Borkh. | Rosaceae | apple | fruit | 740 | RRV | 200-300 | toxic |
| *Malus domestica* Borkh. | Rosaceae | apple | fruit | 1781 | RRV | 400-500 | - |
| *Mangifera indica* L. | Anacardiaceae | mango | fruit | 1900 | RRV | 400-500 | 646,7 |
| *Melia azedarach* L. | Meliaceae | chinaberry | fruit | 973 | RRV | 400-500 | 340,2 |
| *Melissa officinalis L.* ssp. officinalis | Lamiaceae | lemon balm | leaves | 697 | RRV | 400-500 | 404,3 |
| *Melissa officinalis L.* ssp. officinalis | Lamiaceae | lemon balm | leaves | 697 | RRV | 300-400 | 400,9 |
| *Mentha haplocalyx* Briq. | Lamiaceae | filed mint | herb | 8682 | RRV | 400-500 | - |
| *Mentha haplocalyx* Briq. | Lamiaceae | filed mint | herb | 8682 | RRV | 200-300 | 558,6 |
| *Mentha piperita* L. | Lamiaceae | peppermint | herb | 722 | RRV | 400-500 | 574,3 |
| *Moringa oleifera* Lam. | Moringaceae | horseradish tree | fruit/leaf | 11604 | RRV | 400-500 | - |
| *Morus alba* L. | Moraceae | white mulberry | fruit | 8182 | RRV | 400-500 | 644,9 |
| *Morus alba* L. | Moraceae | white mulberry | leaf | 8183 | RRV | 400-500 | 479,1 |
| *Morus alba* L. | Moraceae | white mulberry | leaf | 8183 | RRV | 400-500 | 566,4 |
| *Myristica fragrans* Houtt. | Myristicaceae | nutmeg | seed | 8391 | RRV | 400-500 | 848,4 |
| *Nelumbo nucifera* Gaertn. | Nelumbonaceae | sacred water lotus | fruit | 65 | RRV | 400-500 | 145,0 |
| *Nelumbo nucifera* Gaertn. | Nelumbonaceae | sacred water lotus | fruit | 65 | RRV | 100-200 | 148,5 |
| *Nelumbo nucifera* Gaertn. | Nelumbonaceae | sacred water lotus | leaves | 66 | RRV | 400-500 | - |
| *Nelumbo nucifera* Gaertn. | Nelumbonaceae | sacred water lotus | seed | 410 | RRV | 400-500 | 833,1 |
| *Nigella sativa* L. | Ranunculaceae | black cumin | seed | 11611 | RRV | 400-500 | - |
| *Ocimum basilicum* L. | Lamiaceae | basil | herb | 11859 | RRV | 400-500 | 1022,2 |
| *Ocimum gratissimum* Forssk. | Lamiaceae | clove basil | herb | 11669 | RRV | 400-500 | 570,0 |
| *Olea europaea* L. var. europaea | Oleaceae | olive | leaves | 706 | RRV | 400-500 | 254,9 |
| *Olea europaea* L. var. europaea | Oleaceae | olive | leaves | 706 | RRV | 200-300 | 326,2 |
| *Origanum majorana* L. | Lamiaceae | marjoram | flower essential oil | 8541 | RRV | 400-500 | - |
| *Oryza sativa* L. | Poaceae | rice | herb/seed | 2271 | RRV | 400-500 | - |
| *Perilla frutescens* ( L. ) Britton | Lamiaceae | beefsteak plant | unknown | 502 | RRV | 300-400 | 911,0 |
| *Perilla frutescens* ( L. ) Britton | Lamiaceae | beefsteak plant | seed | 1905 | RRV | 400-500 | - |
| *Perilla frutescens* ( L. ) Britton | Lamiaceae | beefsteak plant | leaf | 8238 | RRV | 400-500 | - |
| *Phaseolus vulgaris* L. | Leguminosae | pinto bean | fruit | 1923 | RRV | 400-500 | 662,6 |
| *Phragmites communis* Trin. | Poaceae | common reed | rhizome | 8352 | RRV | 400-500 | toxic |
| *Phyllanthus emblica* ( L. ) Gaertner | Euphorbiaceae | emblic | fruit | 264 | RRV | 400-500 | toxic |
| *Phyllanthus emblica* ( L. ) Gaertner | Euphorbiaceae | emblic | fruit | 1058 | RRV | 400-500 | toxic |
| *Physalis peruviana* L. | Solanaceae | cape gooseberry | fruit | 25475 | RRV | 400-500 | 819,5 |
| *Phytolacca americana* L. | Phytolaccaceae | american pokeweed | root | 8404 | RRV | 400-500 | - |
| *Platycodon grandiflorum* ( Jacq. ) A. DC. | Campanulaceae | balloon flower | root | 1832 | RRV | 400-500 | 691,6 |
| *Prunella vulgaris* L. | Lamiaceae | selfheal | flower | 1877 | RRV | 400-500 | contaminated |
| *Prunus armeniaca* L. | Rosaceae | apricot | seed | 8440 | RRV | 400-500 | - |
| *Psidium guajava* L. | Myrtaceae | guava | fruit | 1132 | RRV | 400-500 | - |
| *Punica granatum* L. | Lythraceae | pomegranate | fruit | 370 | RRV | 400-500 | toxic |
| *Punica granatum* L. | Lythraceae | pomegranate | fruit | 370 | RRV | 200-300 | toxic |
| *Punica granatum* L. | Lythraceae | pomegranate | fruit | 667 | RRV | 400-500 | toxic |
| *Punica granatum* L. | Lythraceae | pomegranate | fruit | 667 | RRV | 200-300 | toxic |
| *Punica granatum* L. | Lythraceae | pomegranate | fruit peel | 8413 | RRV | 400-500 | - |
| *Raphanus sativus* L. ssp *sativus* | Brassicaceae | radish | unknown | 918 | RRV | 400-500 | 685,4 |
| *Raphanus sativus* L. ssp *sativus* | Brassicaceae | radish | fruit | 8345 | RRV | 400-500 | - |
| *Rhus succedanea* L. | Anacardiaceae | wax tree | fruit | 25487 | RRV | 400-500 | - |
| *Ribes nigrum* L. | Grossulariaceae | black currant | fruit | 8489 | RRV | 400-500 | 526,1 |
| *Ribes nigrum* L. | Grossulariaceae | black currant | fruit | 8489 | RRV | 400-500 | 713,6 |
| *Rosmarinus officinalis* L. | Lamiaceae | rosemary | leaf | 835 | RRV | 400-500 | toxic |
| *Rosmarinus officinalis* L. | Lamiaceae | rosemary | leaf | 835 | RRV | 200-300 | toxic |
| *Rosmarinus officinalis* L. | Lamiaceae | rosemary | unknown | 854 | RRV | 400-500 | 478,0 |
| *Rosmarinus officinalis* L. | Lamiaceae | rosemary | unknown | 854 | RRV | 400-500 | 506,0 |
| *Salvia officinalis* L. ssp minor | Lamiaceae | sage | leaf | 1126 | RRV | 400-500 | 645,1 |
| *Satureja montana* L. | Lamiaceae | winter savory | herb | 11860 | RRV | 400-500 | - |
| *Saussurea lappa* ( Decne. ) C.B.Clarke | Asteraceae | costus | root | 25490 | RRV | 400-500 | toxic |
| *Smilax glabra* Roxb. | Smilacaceae | sarsaparilla | rhizome | 8203 | RRV | 400-500 | 972,8 |
| *Smilax glabra* Roxb. | Smilacaceae | sarsaparilla | rhizome | 8429 | RRV | 400-500 | 541,2 |
| *Smilax glabra* Roxb. | Smilacaceae | sarsaparilla | rhizome | 8663 | RRV | 400-500 | - |
| *Solanum torvum* Sw. | Solanaceae | pea eggplant | fruit/seed | 976 | RRV | 400-500 | - |
| *Spinacia oleracea* L. | Chenopodiaceae | spinach | leaf | 25550 | RRV | 400-500 | - |
| *Stevia rebaudiana* Bertoni | Asteraceae | sweet honey leaf | unknown | 808 | RRV | 400-500 | 591,7 |
| *Stevia rebaudiana* Bertoni | Asteraceae | sweet honey leaf | herb | 2074 | RRV | 400-500 | - |
| *Stevia rebaudiana* Bertoni | Asteraceae | sweet honey leaf | unknown | 11977 | RRV | 100-200-300 | 451,9 |
| *Stevia rebaudiana* Bertoni | Asteraceae | sweet honey leaf | unknown | 11977 | RRV | 400-500 | 576,6 |
| *Syzygium aromaticum* ( L. ) Merr. & L.M.Perry | Myrtaceae | clove | herb | 25611 | RRV | 400-500 | 600,1 |
| *Theobroma cacao* L. | Sterculiaceae | cacao | fruit/seed | 380 | RRV | 400-500 | - |
| *Theobroma cacao* L. | Sterculiaceae | cacao | seed | 400 | RRV | 400-500 | - |
| *Trichosanthes kirilowii* Maxim. | Cucurbitaceae | chinese cucumber | fruit | 8124 | RRV | 400-500 | - |
| *Urtica dioica* L. | Urticaceae | stinging nettle | root | 526 | RRV | 400-500 | 486,3 |
| *Urtica dioica* L. | Urticaceae | stinging nettle | root | 526 | RRV | 400-500 | 246,6 |
| *Urtica dioica* L. | Urticaceae | stinging nettle | root | 526 | RRV | 200-300 | 93,7 |
| *Urtica dioica* L. | Urticaceae | stinging nettle | root | 526 | RRV | 100-200 | 207,1 |
| *Urtica dioica* L. | Urticaceae | stinging nettle | root | 526 | RRV | 300-400 | 395,2 |
| *Vaccinium myrtillus* L. | Ericaceae | bilberry | fruit | 746 | RRV | 400-500 | toxic |
| *Vaccinium myrtillus* L. | Ericaceae | bilberry | fruit | 746 | RRV | 200-300 | 774,0 |
| *Vaccinium vitis-idaea* L. | Ericaceae | mountain cranberry | unknown | 25222 | RRV | 400-500 | - |
| *Valeriana jatamansi* D.Don | Valerianaceae | indian valerian | root | 1719 | RRV | 400-500 | 740,4 |
| *Valeriana jatamansi* D.Don | Valerianaceae | indian valerian | root | 1748 | RRV | 400-500 | - |
| *Vernonia amygdalina* Delile | Asteraceae | bitterleaf | leaf | 11942 | RRV | 400-500 | 673,6 |
| *Viola yedoensis* Makino | Violaceae | chinese violet | herb | 8237 | RRV | 400-500 | 641,1 |
| *Zea mays* L. ssp *mays* | Poaceae | corn | unknown | 933 | RRV | 400-500 | 941,9 |
| *Zea mays* L. ssp *mays* | Poaceae | corn | pistil | 8669 | RRV | 400-500 | - |
| *Zea mays* L. ssp *mays* | Poaceae | corn | maydis | 11828 | RRV | 400-500 | - |
| *Zingiber officinale* Roscoe | Zingiberaceae | ginger | root | 220 | RRV | 400-500 | 488,7 |
| *Zingiber officinale* Roscoe | Zingiberaceae | ginger | root | 220 | RRV | 400-500 | 508,0 |
