# Supplementary material for: An evaluation of the inhibitory effects against rotavirus infection of edible plant extracts
Source: Virol J. 2012 Jul 26;9:137. doi: 10.1186/1743-422X-9-137 (PMC3439294; doi:10.1186/1743-422X-9-137)
Supplement: Additional file 2 — Appendix 2. List of tested pure components. [file 1743-422X-9-137-S2.doc]

Appendix 2: List of tested pure components

| **Compound** | **Brand** | **Purity** | **Tested conc. µM** | **IC50 (µM)** | **Description** |
| --- | --- | --- | --- | --- | --- |
| (±)-L-Alliin | Fluka 74264 | ≥90% (HPLC) | 50-100 | - | Sulfur-containing amino acid that is converted to allicin by alliinase |
| Apigenin-7-O-glucoside | Indofine 021004S | >99% (HPLC) | 50-100 | 150,6 | flavone |
| Caffeic acid | Sigma-Aldrich C0625 | ≥98% (HPLC) | 50-100 µM | - | tannin; consists of both phenolic and acrylic functional groups |
| Chrysoeriol | Indofine 021104S | ≥99% (HPLC) | 50-100 | - | yellow crystalline flavone pigment |
| Citral | Sigma-Aldrich W230316 | ≥95% (FCC) | 50-100 µM | - | (a mixture of) a pair of terpenoids |
| Geraniol | MP Biomedicals 157184 | 98% | 50-100 µM | - | monoterpenoid and an alcohol |
| d-Limonene | MP Biomedicals 155234 | 96.9% | 50-100 µM | - | cyclic terpene |
| Diosmin | MP Biomedicals 157857 | 95% | 50-100 | - | naturally occurring flavonoid glycoside or derived from hesperidin |
| (-)- Epicatechin | Sigma-Aldrich E1753 | ≥90% (HPLC) | 50-100 | - | flavanol |
| *trans*-Ferulic acid | Sigma-Aldrich W518301 | ≥99% | 50-100 | - | hydroxycinnamic acid found naturally in plant cell walls, leaves and seeds |
| Foscarnet | Sigma-Aldrich P6801 | unknown | 100-150-200 | - | phosphonic acid derivative |
| 18ß-glycyrrhetinic acid | Sigma-Aldrich G1010 | 97% | 50-45-40-35-30 | 45,9 | pentacyclic triterpenoid derivative of glycyrrhizic acid |
| Hesperidin | MP Biomedicals 101933 | ~80% | 50-100 | - | flavanone glycoside |
| Kaempferol | Indofine K102 | 99% | 50-100 | - | flavonol |
| Luteolin | Indofine 021125S | >99% (HPLC) | 50-100 | 115,8 | flavone |
| Luteolin-7-O-glucoside | Indofine 020053 | >90% (HPLC) | 50-100 | 277,1 | flavone |
| Naringin | MP Biomedicals 155802 | 100,18% | 50-100 | - | flavanone glycoside |
| Oleanolic acid | Sigma-Aldrich O5504 | ≥97% | 50-100 | - | naturally occurring triterpenoid |
| Quercetin-3-O-glucopyranoside | Indofine 020074 | ≥90% (HPLC) | 50-100 | +/- | plant-derived flavonol |
| Resveratrol | Sigma-Aldrich R5010 | ≥99% (GC) | 1-5-10 µg/ml | - | stilbenoid, a type of natural phenol |
| Rutin Trihydrate | MP Biomedicals 102824 | >95% | 50-100 | - | flavonoid glycoside |
| ß-Sitosterol | MP Biomedicals 102886 | 92.8% (total steroids) | 50-100 µM | - | composition: ß-Sitosterol ~40-60%; campesterol ~20-40%; Stigmasterol ~ 5% |
| Vitexin | Indofine 021232S | ≥99% (HPLC) | 50-100 | 128,9 | apigenin flavone glucoside |
| Xanthophyll | MP Biomedicals 103292 | >6% in corn base oil | 50-100 µM | 251,2 | yellow carotenoid pigment |
